# Journal quartiles and article-level citation performance: a single-institution case study in academic dentistry

**DOI:** 10.3389/frma.2026.1894451

**Published:** 2026-09-07

**Authors:** Andy Wai Kan Yeung

**Affiliations:** Oral and Maxillofacial Radiology, Applied Oral Sciences and Community Dental Care, Faculty of Dentistry, University of Hong Kong, Hong Kong, Hong Kong SAR, China

**Keywords:** citation analysis, dentistry research, journal quartiles, normalized citation impact (CNCI), research evaluation, scholarly publishing

## Abstract

Journal quartiles derived from the journal citation reports are widely used for research evaluation, yet empirical evidence on their relationship with article-level normalized citation impact within specific disciplines remained limited. By examining this relationship using article-level indicators, the study explicitly addresses how journal quartiles function in institutional research evaluation practice, rather than as standalone bibliometric descriptors. This study analyzed 1,180 publications (2015–2024) from a research-intensive dental school to examine how journal quartile related to category normalized citation impact (CNCI). Descriptive analyses demonstrated a strong and orderly gradient in CNCI and raw citation counts from Q4 to Q1. Multivariable Gamma regression with log link and negative binomial models showed that quartile effects persist after adjusting for collaboration type, authorship, document type, and publication year. These findings aligned with cross-field scientometric evidence showing that higher-quartile journals attract disproportionate citation attention. The results extended prior journal-level analyses by showing a strong adjusted association between quartile and article-level normalized performance on aggregate. The study provided discipline-specific evidence relevant to institutional research strategy and publication planning. Future work could assess whether similar quartile gradients appear across other academic healthcare or biomedical subject areas.

## 1 Introduction

Over the past decade, major international initiatives such as the San Francisco declaration on research assessment (DORA), the Leiden Manifesto, and the coalition for advancing research assessment (CoARA) have explicitly challenged the use of journal-based indicators, including impact factor and derived quartiles, as proxies for research quality at the level of individual articles or researchers. These developments have shifted the research evaluation landscape toward greater emphasis on article-level assessment and responsible use of metrics. However, despite these policy shifts, journal-based indicators remain widely used in institutional practice in research performance evaluation, funding allocation, and institutional benchmarking (Vîiu and Păunescu, 2021). Among these, journal quartiles derived from the journal citation reports (JCR) are widely used as a proxy for journal prestige and selectivity, and increasingly as default indicators of research “quality” in academic assessment systems. In many countries and disciplines, publication in first-quartile (Q1) journals is explicitly incentivized through promotion criteria, performance-based funding schemes, and institutional ranking frameworks (Vîiu and Păunescu, 2021). Despite persistent methodological critiques of journal-level metrics, quartiles remain deeply embedded in academic decision-making because of their simplicity, comparability within subject categories, and perceived objectivity.

From a scientometric perspective, the widespread reliance on journal quartiles raises an important empirical question: to what extent does journal quartile relate to the citation impact of individual articles, after accounting for disciplinary differences, publication age, and collaboration patterns? While it is well established that citation distributions are highly skewed and that journal-level metrics poorly predict the impact of individual papers (Bornmann and Leydesdorff, 2017), a growing body of evidence nonetheless suggests that higher-tier journals confer systematic visibility advantages that may shape downstream citation outcomes through mechanisms such as selective editorial practices, broader readership, and cumulative advantage effects (Wang, 2014; Teixeira Da Silva, 2021).

Large-scale cross-disciplinary studies have repeatedly demonstrated that Q1 journals capture a disproportionate share of citations relative to their output. For example, Miranda and García-Carpintero showed that across 25 research areas, publications in Q1 journals received substantially more citations on average than those in lower quartiles, although the magnitude of this advantage varied considerably by field (Miranda and Garcia-Carpintero, 2019). Such findings highlight that quartile effects are real but potentially field-dependent. However, most existing analyses operate either at the journal level, focusing on aggregate citation shares and impact factors, or at the macro disciplinary level, pooling heterogeneous fields with markedly different citation cultures.

Article-level analyses adopting field-normalized citation indicators provide a more appropriate framework for assessing the contribution of publication venue to citation performance. Metrics such as category normalized citation impact (CNCI) adjust for subject area, document type, and publication year, enabling more meaningful comparisons across journals and time (Hicks et al., 2015). CNCI is now widely used in institutional analytics platforms such as Clarivate InCites and plays a prominent role in research evaluation exercises at universities and national agencies. Nevertheless, empirical studies examining how CNCI varies systematically across journal quartiles within specific disciplines remain relatively scarce.

Dentistry represents an especially interesting case in this respect. As a clinical and applied biomedical discipline, dentistry spans basic science, translational research, and patient-oriented investigation, each with distinct publication practices and citation dynamics. Prior bibliometric studies in dentistry have largely focused on productivity patterns, impact factor rankings of journals, or the citation performance of countries and institutions (Chen et al., 2021; Jin et al., 2022; Yeung and Tsoi, 2025). Journal-level analyses have identified factors such as publication volume, prevalence of systematic reviews, and editorial policies as determinants of a journal's quartile position (Bornmann et al., 2011; Valderrama et al., 2020), yet such studies did not address whether individual articles published in different quartiles exhibit systematically different citation performance once normalized for field and time.

At the same time, scientometric research based on duplicate or near-duplicate publications provided compelling evidence that publication venue itself could meaningfully influence citation trajectories. Comparative analyses by Lariviere and Gingras (2010), as well as Knothe (Knothe, 2006), demonstrated that papers with essentially identical scientific content could receive markedly different citation counts depending on the journals in which they appeared. These studies suggested that when scientific quality is broadly comparable, journal choice may act as an amplifier (or suppressor) of scholarly visibility. However, such evidence has rarely been integrated into discipline-specific analyses using modern normalized indicators such as CNCI.

The present study addressed these gaps by conducting an article-level scientometric analysis of publications produced by the Faculty of Dentistry, The University of Hong Kong, a research-intensive dental school with sustained global visibility. Using a complete institutional corpus extracted from Clarivate InCites covering the period 2015–2024, this study examined whether journal quartile was associated with systematic differences in normalized citation impact. The study focused primarily on CNCI as the main outcome measure and complemented this analysis with models of raw citation counts to enhance interpretability for readers less familiar with normalized indicators.

Despite the widespread use of journal quartiles in promotion criteria, institutional benchmarking, and research management, there is limited empirical evidence based on article-level, field-normalized indicators on what quartile placement actually corresponds to in terms of realized citation performance. This gap complicates the responsible interpretation of quartiles in research evaluation practice, particularly when they are applied to individual publications or researchers. Addressing this issue requires moving beyond journal-level metrics to examine how quartiles translate into article-level outcomes under realistic evaluative conditions.

Specifically, this study addressed three research questions. First, is there a monotonic gradient in CNCI across journal quartiles (Q4 to Q1) in dentistry publications? Second, does any observed quartile gradient persist after adjusting for collaboration type, authorship scale, document type, publication year, and manuscript length? Third, do analyses based on raw citation counts yield patterns consistent with those observed for CNCI? By answering these questions, the study contributes discipline-specific empirical evidence to ongoing debates on the role of journal quartiles in research evaluation and offers insights relevant to publication strategy within academic dentistry. At the same time, this study was designed to quantify adjusted associations between journal quartile and article-level citation impact within one institutional corpus, but did not identify a causal effect of journal placement.

## 2 Methods

All analyses were conducted at the paper level using publication records exported on 20 March 2026 from InCites Benchmarking & Analytics online database (Clarivate Analytics), restricted to Articles and Reviews published between 2015 and 2024 in journals classified under Dentistry, Oral Surgery & Medicine. Only outputs attributed to HKU Dentistry were included based on the InCites institutional and departmental filtering.

The primary independent variable was journal quartile (Q1–Q4), defined according to the best JIF quartile within the Dentistry category as assigned by InCites. Quartiles were treated as an ordered factor (Q4 < Q3 < Q2 < Q1), reflecting increasing journal prestige.

The primary outcome was category normalized citation impact (CNCI), a continuous normalized citation metric representing the ratio of observed citations to expected citations for papers of the same category (subject area and document type) and publication year. The secondary outcome was times cited (TC), representing raw citation counts accumulated up to the 2026 InCites index date.

Pre-specified covariates are listed below:

- Publication year, modeled as a fixed effect to account for temporal differences in citation opportunity.
- Document type (Article vs. Review).
- Collaboration type, dichotomized as International vs. Domestic based on InCites' collaboration categories.
- Author count, parsed from author lists; modeled as natural log(author count) to reflect diminishing marginal citation gains with increasing team size.
- Page count, derived from page ranges; modeled as natural log(page count) to reduce skew and approximate linearity.

Descriptive summaries of CNCI across quartiles included counts, medians, means, and dispersion metrics were reported. The Jonckheere-Terpstra (JT) test was used to evaluate if there was a monotonic trend in CNCI across the ordered quartiles (Q4 → Q1). To further assess differences between adjacent quartiles, *post hoc* pairwise comparisons of quartile groups were performed in SPSS using rank-based nonparametric pairwise tests (one-sided). Bonferroni correction was applied to account for multiple comparisons (Q1 vs. Q2, Q2 vs. Q3, Q3 vs. Q4). Effect sizes for adjacent comparisons were summarized using $r=\;|Z|/\sqrt{N}$, where *N* is the total number of observations in the two groups being compared.

To estimate adjusted differences in CNCI between quartiles, two Gamma regressions with a log link were fitted. The first model included all papers with CNCI > 0 and valid page count (*N* = 812). The second model included all papers with CNCI > 0 (*N* = 1,153). CNCI was treated as a continuous dependent variable, with Q4 as the reference category. The model included all covariates listed above and year fixed effects. The second model excluded page count as a covariate, in order to include more papers. CNCI was not adjusted for publication age, as expected citation denominators in the metric inherently normalize for publication year.

As a secondary analysis, TC was also analyzed. Because TC are discrete, right-skewed, and over-dispersed, it was modeled using a negative binomial generalized linear model (NB-GLM) (*N* = 826). To correct for differential citation exposure, the model incorporated an offset term equal to log(publication age in years), yielding adjusted incidence rate ratios (IRRs) relative to Q1. The same covariates and publication-year fixed effects used in the first Gamma model were applied here.

Statistical tests were performed in SPSS 28.0 (IBM, NY, USA). Figures and ancillary processing were performed in Python. Statistical results were significant if *p* < 0.05.

## 3 Results

**Figure 1:**
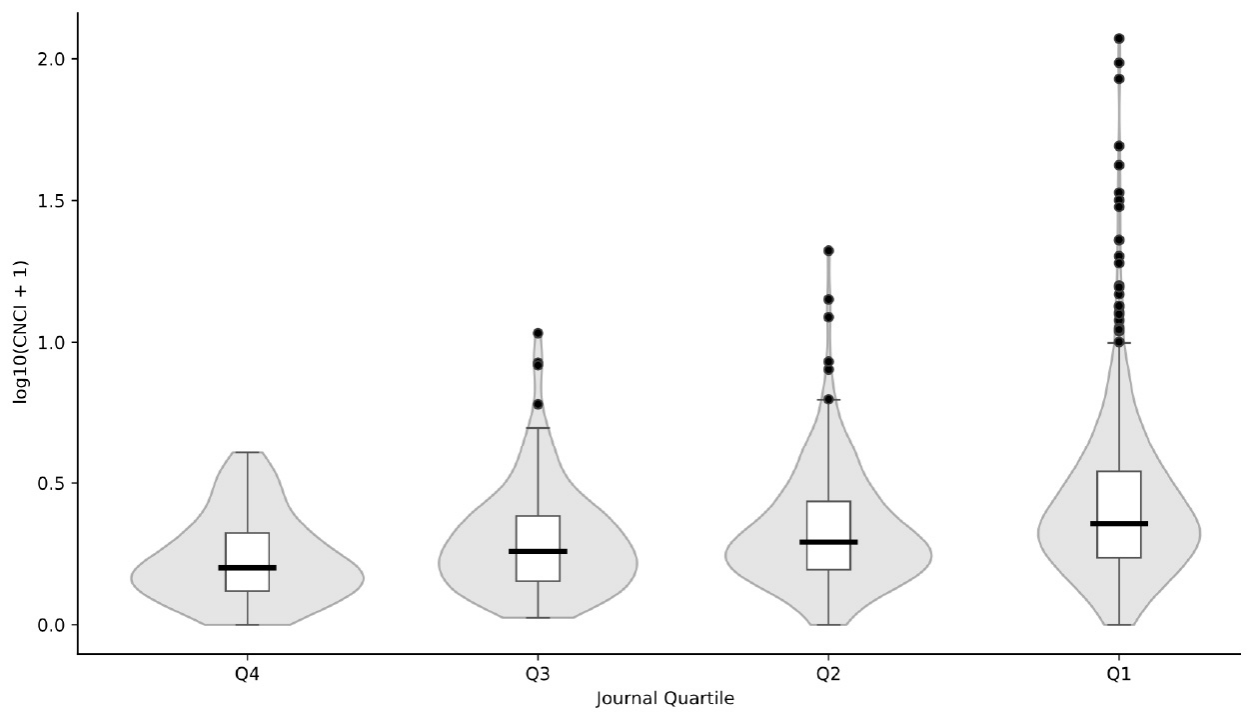
Distribution of CNCI values across journal quartiles (Q4–Q1) for HKU Dentistry publications between 2015–2024. Q1 papers show higher median CNCI and a pronounced upper tail. Because CNCI was right-skewed, y-axis is presented in a log scale, improving separation of medians across quartiles.

A total of 1,180 HKU Dentistry publications from 2015–2024 were included (Supplementary Material). CNCI values showed an upward monotonic trend across quartiles (T_JT_ = 151309.5, *z* = 9.168, *p* < 0.001), with Q1 papers exhibiting the highest median and mean CNCI, followed by Q2, Q3, and Q4 in descending order (Table 1). This pattern was evident in both the distributional spread and central tendency: Q1 papers displayed a noticeably longer upper tail and higher concentration of high-impact values (Figure 1).

**Table 1:** Descriptive statistics of CNCI of papers published in different journal quartiles (*n* = 1180).

Quartile	Count	Median	IQR (Q1–Q3)	Mean	SD
Q1	684	1.27	0.52–3.06	2.71	7.53
Q2	266	0.96	0.39–1.82	1.45	1.88
Q3	143	0.81	0.34–1.33	1.20	1.35
Q4	87	0.59	0.29–0.95	0.82	0.71

Pairwise comparisons of adjacent quartiles further illustrated this gradient (Table 2). Effect sizes indicated that CNCI differences were small between adjacent groups, and the difference between Q2 and Q3 was not significant after Bonferroni correction.

**Table 2:** *Post-hoc* pairwise comparison results between adjacent quartiles.

Comparison	Standardized test statistic (Z)	Effect size *r*	*P* (Bonferroni corrected)
Q1 vs. Q2	5.07	0.16	< 0.001
Q2 vs. Q3	1.82	0.09	0.102
Q3 vs. Q4	2.58	0.17	0.015

After adjustment for document type, collaboration, author team size, page count, and publication year, the quartile gradient remained strong. Relative to Q4, the estimated adjusted CNCI ratios were approximately 2.96 for Q1, 1.69 for Q2, and 1.21 for Q3, with Q1 and Q2 (but not Q3) being significantly higher (Table 3). These differences indicate that, even after controlling for a broad set of covariates, journal quartile was still associated with article-level CNCI. Second model that excluded page count as a covariate showed a similar pattern, with Q1, Q2, and Q3 all significant (Table 4).

**Table 3:** Results of first Gamma regression with a log link, with CNCI treated as a continuous dependent variable, and Q4 as the reference category.

Term	B	Adjusted CNCI ratio, exp(B)	95% CI for exp(B)	*P* (Bonferroni corrected)
Q1 vs. Q4	1.084	2.96	2.33–3.75	< 0.001
Q2 vs. Q4	0.522	1.69	1.31–2.17	< 0.001
Q3 vs. Q4	0.188	1.21	0.92–1.58	0.171

The model included year fixed effects and covariates: document type, collaboration type, log(author count), and log(page count).

**Table 4:** Results of second Gamma regression with a log link, with CNCI treated as a continuous dependent variable, and Q4 as the reference category.

Term	B	Adjusted CNCI ratio, exp(B)	95% CI for exp(B)	*P* (Bonferroni corrected)
Q1 vs. Q4	1.105	3.02	2.42–3.77	< 0.001
Q2 vs. Q4	0.598	1.82	1.44–2.30	< 0.001
Q3 vs. Q4	0.402	1.49	1.16–1.93	0.002

The model included year fixed effects and covariates: document type, collaboration type, log(author count).

The secondary analysis of raw citation counts showed a consistent pattern. When modeling TC with adjustment for publication age and the abovementioned covariates, the expected citation rate for Q1 papers was markedly higher than for lower quartiles (Table 5). Specifically, the estimated incidence rate ratios were 0.56 for Q2, 0.42 for Q3, and 0.34 for Q4 compared with Q1. These values indicate that Q4 papers accumulated citations at roughly one-third the rate of Q1 papers, with Q2 and Q3 papers showing intermediate citation rates.

**Table 5:** Negative binomial generalized linear model (NB-GLM) results.

Term	IRR	95% confidence interval	*P*
Q2	0.56	0.47–0.68	< 0.001
Q3	0.42	0.34–0.52	< 0.001
Q4	0.34	0.26–0.44	< 0.001

To correct for differential citation exposure, the model incorporated an offset term equal to log(publication age in years), yielding adjusted incidence rate ratios (IRRs) relative to Q1. The same covariates and publication-year fixed effects used in the first Gamma model were applied here.

Taken together, the descriptive patterns, adjusted CNCI differences, and adjusted citation-rate ratios all converge on the same conclusion: journal quartile is strongly and consistently associated with citation performance, with Q1 > Q2 > Q3 > Q4 across both normalized and raw citation indicators.

## 4 Discussion

Within this institutional corpus, this study provides a clear and intuitive message: publication venue was strongly associated with citation performance. Across nearly 1,200 HKU Dentistry papers published over a decade, one could observe a strong and consistent gradient in normalized citation performance (Q1 > Q2 > Q3 > Q4) that persisted even after controlling for collaboration, author count, document type, and publication year. In other words, after adjustment for measured covariates such as collaboration type and author count, publishing in a higher-quartile journal systematically associated with higher impact.

This pattern is not surprising in the broader scientometric context, but it has rarely been demonstrated so cleanly within dentistry. Previous work has long suggested that journal prestige is associated with increased visibility, citation advantages, and cumulative-advantage mechanisms (sometimes described as “the Matthew effect”), where papers published in high-status venues attract more readers and citations simply because of their placement (Wang, 2014; Yeung et al., 2020; Teixeira Da Silva, 2021). Besides, in a large cross-disciplinary analysis of 25 research areas, Miranda and Garcia-Carpintero (2019) demonstrated that publications in Q1 journals received, on average, more than twice as many citations as those in Q2 journals, with the Q1-to-Q2 ratio varying between 0.9 and 6.1 across fields (Miranda and Garcia-Carpintero, 2019). Although such patterns have been reported in medicine and the life sciences, field-specific analyses in dentistry are limited. By using CNCI, a field- and year-normalized metric that is widely accepted in research evaluation frameworks, the current findings reinforce that journal quartile remained associated with CNCI in adjusted models.

These findings may be informative for similar research-intensive dental institutions. If future studies could generalize these findings with multi-institutional replications, the following policy implications may be considered. First, they validate the intuition held by many researchers and department heads: these findings provide empirical support for encouraging faculty members to consider higher-quartile journals, while recognizing the limitations and contextual nature of quartile-based indicators. Second, the findings offer a quantitative foundation for mentoring early-career staff on where to direct their submissions, especially in subfields with multiple competing journals across quartiles. Finally, the evidence supports strategic planning within the faculty's research office, where publication venue choices feed into downstream metrics used in global rankings such as QS and THE.

At the same time, these findings should not be interpreted as a call for “Q1 at all costs,” but rather as evidence that quartiles should be used as contextual evaluative signals whose limitations are explicitly acknowledged in formal assessment procedures. High-quartile journals are often more selective, and subfield diversity means that Q2 or Q3 journals can still play an important role in knowledge dissemination (Kosyakov and Pislyakov, 2024; Paul, 2024). Journal placement may be associated with greater visibility and citation accumulation, although causality cannot be inferred. This is evidenced by prior studies such as Lariviere and Gingras (2010) and Knothe (2006) showing that papers with essentially identical scientific content receive markedly different citation counts depending on the journals in which they appear (Knothe, 2006; Lariviere and Gingras, 2010). Quartiles should therefore be interpreted as coarse evaluative signals rather than deterministic quality thresholds, particularly when applied to individual publications or researchers.

This study has several limitations. First, although 1,180 papers were included in descriptive and non-parametric analyses, the complete-case Gamma model (first model) used 812 papers only, and the second Gamma model used 1,153 papers, leaving 27 papers with CNCI = 0 excluded as Gamma regression requires positive outcomes. Also, many contemporary journals use article numbers instead of traditional pagination. This issue is increasingly common as publishers move toward continuous online publication. The phenomenon of missing page numbers appears unrelated to citation performance or journal quartile; it reflects publishing format rather than scientific quality. Second, this is a single-institution case study, though covering a world-leading dental school with a large and diverse publication portfolio. While this limits generalizability in a strict statistical sense, it also affords advantages: because the analysis covers an entire faculty's output over ten years, the dataset is internally consistent, avoids sampling bias, and reflects the realities of research strategy within a competitive dental school. Third, CNCI is not a perfect metric, as it does not adjust for topic-level citation idiosyncrasies (e.g., hot topics vs. niche areas). However, it remains one of the most defensible metrics available for article-level, cross-field comparisons and is commonly used in strategic planning in research-intensive universities. Finally, the existing scientometric literature on dentistry often focuses on institutional h-indices, impact factor rankings, or the output of particular research themes. Few have examined nearly a decade of article-level citation performance using CNCI, nor have they linked journal quartile choice to normalized citation outcomes using robust statistical models. Thus, the current analytic approach and findings contribute meaningfully to the evidence base on dental research performance and may serve as a practical reference for other universities seeking data-driven insights into publication strategy.

Still, readers should be aware that the present findings do not resolve the normative debate surrounding the use of journal quartiles in research evaluation. Instead, they highlight a tension between empirical predictiveness and normative critiques. While quartile placement is associated with citation performance on average, the persistence of this relationship does not justify its uncritical use in evaluation frameworks, particularly given well-documented limitations and concerns about fairness, transparency, and field variations. As the study is observational, the adjusted quartile differences reported here should not be interpreted as evidence that publication in a higher-quartile journal by itself causes higher citation impact. Unmeasured factors such as topic salience, manuscript quality, author reputation, and funding may influence both journal placement and subsequent citations.

## 5 Conclusion

This study offers empirical evidence that journal quartile gradient persisted in adjusted models. Across nearly 1,200 publications from a leading dental school, a pronounced and orderly gradient in CNCI and citation counts from Q4 to Q1 was observed. Notably, these differences persisted after adjusting for collaboration patterns, authorship, document type, and publication year, underscoring that the citation advantage of Q1 journals cannot be attributed solely to research team structure or publication characteristics. Instead, journal quartile may reflect a combination of venue-related visibility and unmeasured selection processes. Readers should note that the results are associational rather than causal.

## Data Availability

The original contributions presented in the study are included in the article/Supplementary material, further inquiries can be directed to the corresponding author.
